# An overview and single-arm meta-analysis of immune-mediated adverse events following COVID-19 vaccination

**DOI:** 10.3389/fphar.2024.1308768

**Published:** 2024-06-12

**Authors:** Donghua Yang, Jinhui Tian, Caiyi Shen, Qin Li

**Affiliations:** ^1^ Department of Public Health and Hospital Infection Management, Qinghai University Affiliated Hospital, Xining, China; ^2^ Evidence-Based Medicine Center, School of Basic Medical Sciences, Lanzhou University, Lanzhou, China; ^3^ The First Clinical Medical College of Lanzhou University, Lanzhou, China; ^4^ Hunan University of Medicine, Huaihua, China

**Keywords:** COVID-19 vaccination, immune-mediated adverse events, overview, myocarditis, thrombosis

## Abstract

**Background:**

We conducted an overview to assess immune adverse effects associated with the COVID-19 vaccine, guiding safer choices and providing evidence-based information to clinicians.

**Methods:**

Forty-three studies on adverse effects of vaccines were reviewed from PubMed, Embase, and Web of Science. Single-arm meta-analyses estimated summary effects, incidence, presentation, etc. An overview using single-arm meta-analysis and reported the findings following the guidelines outlined in the ‘Preferred Reporting Items for Systematic Reviews and Meta-analyses (PRISMA) specifically focusing on myocarditis and thrombosis. After screening 2,591 articles, 42 studies met the inclusion criteria. Methodological quality was evaluated using AMSTAR 2. Disagreements were resolved via consensus. Data analysis utilized a random-effects model in R software to estimate incidence rates of selected adverse events.

**Results:**

After removing 1,198 duplicates and screening out irrelevant articles from a total of 2,591, we included 42 studies. Adverse reactions to vaccinations include myocarditis, thrombosis, skin reactions, GBS, etc. thrombosis and myocarditis are the most dangerous diseases associated with vaccination. Myocarditis occurred in 6% of Vector vaccine recipients, compared to 61% of mRNA vaccine recipients. Thrombosis was more common after Vector vaccination (91%) than after mRNA vaccination (9%). Furthermore, eight studies conducted anti-PF4 antibody tests and yielded a positivity rate of 67%. Meta-analysis showed that among all patients with Vaccine-induced Thrombotic Thrombocytopenia, cerebral venous sinus thrombosis occurred in 66%, and intracranial hemorrhage occurred in 43%. The rates of deep vein thrombosis and pulmonary thromboembolism in vaccinated patients were 13% and 23%, respectively, with a pooled case fatality rate of 30%.

**Conclusion:**

The results of this overview indicate the majority of adverse reactions are self-limiting and require minimal intervention, while rare occurrences such as myocarditis and thrombosis pose a potentially fatal threat.

## Introduction

Vaccines have played a pivotal role in combating the SARS-CoV-2 pandemic, offering a crucial defense against severe infections (Greinacher et al., 2021). The landscape of COVID-19 vaccines encompasses diverse formulations, including mRNA vaccines like BNT162b2 (Pfizer-BioNTech) and mRNA-1273 (Moderna), adenovirus vector vaccines such as ChAdOx1 nCov-19 (AstraZeneca) and the Johnson and Johnson/Janssen vaccine, as well as recombinant protein and traditional vaccines like CoronaVac and Covaxin/BBV152 (Greinacher et al., 2021; Schulz et al., 2021; Kim et al., 2022a; Kolahchi et al., 2022).

While the benefits of vaccination are well-acknowledged, it is crucial to also recognize and address the potential adverse reactions that may follow immunization. Like any medical intervention, vaccines are not without their risks, necessitating a careful evaluation of their benefits against possible undesirable effects. This is particularly relevant in the wake of the COVID-19 pandemic, as a significant body of research has been dedicated to investigating the adverse outcomes associated with SARS-CoV-2 vaccines. These investigations range from individual case reports to comprehensive systematic reviews and cover a variety of adverse events including myocarditis, thrombotic events, skin reactions, dermatologic side effects, late-onset myasthenia gravis, and hypermetabolic lymph nodes (ElSawi and Elborollosy, 2022; Rosenblum et al., 2022; Yuniar et al., 2022).

Although most of these reactions are mild and temporary, certain conditions such as myocarditis and thrombotic events have drawn particular scrutiny due to their severity (Barda et al., 2021; Cai et al., 2021; Cordero et al., 2022; Farahmand et al., 2022; Qaderi et al., 2022). Evidence suggests that cases of myocarditis and pericarditis are more likely to occur within 14 days post-vaccination, predominantly following the second dose and especially in younger males (ogrig et al., 2024). These adverse effects have been observed across various vaccine platforms, including ChAdOx1, BNT162b2, and mRNA-1273 (Patone et al., 2022). Furthermore, Thrombosis with Thrombocytopenia Syndrome (TTS) following administration of Vaxzevria and Jcovden vaccines has been reported, with a significant number of cases being fatal (Buoninfante et al., 2022). While the link between TTS and vaccination is still being explored, it emphasizes the importance of ongoing vigilance and research.

Autopsy studies on individuals vaccinated against SARS-CoV-2 are contributing valuable insights. For instance, in one case, myocarditis identified post-vaccination with Comirnaty was considered as a possible vaccine-related cause of death (Schneider et al., 2021). Similarly, Vaccine-induced Thrombotic Thrombocytopenia (VITT) was confirmed in individuals post-vaccination with Vaxzevria and Janssen (Sessa et al., 2021). Another autopsy study in Colombia pointed to sudden cardiac death and pulmonary embolism as significant post-vaccination findings, underscoring the role of autopsies in understanding vaccine-related deaths (Chaves et al., 2023).

To support informed decision-making by the public and offer evidence-based guidance to healthcare professionals, our study employs an overview and meta-analysis to delve into the patient characteristics, symptoms, laboratory findings, and mortality rates associated with adverse reactions to COVID-19 vaccines. By providing a detailed examination of these events, our aim is to balance the understanding of risks and benefits associated with different vaccines and their formulations. This comprehensive approach seeks to inform future vaccine policies and practices, ensuring a safer and more effective vaccine administration strategy.

## Methods

We conducted an overview using single-arm meta-analysis and reported the findings following the guidelines outlined in the ‘Preferred Reporting Items for Systematic Reviews and Meta-analyses (PRISMA). ’We adhered to the PRISMA checklist for this systematic review. However, we chose not to enroll this study in the International Prospective Register of Systematic Reviews (PROSPERO) as we had reservations about disclosing sensitive data in a swiftly developing and significant research domain. However, we had already completed our research protocol prior to commencing the study.

### Inclusion and exclusion criteria

Studies included must meet the following conditions:

1. This study includes systematic reviews written in English.
2. Individuals who have received COVID-19 vaccinations and subsequently experienced adverse reactions, regardless of the vaccine type or dosage.
3. The primary outcomes of interest are myocarditis and thrombosis as adverse effects post-vaccination.

We eliminated the studies that meet any of the following criteria:

1. Research conducted on particular cohorts afflicted with a medical condition as opposed to individuals in decent health.
2. Systematic review studies of Phase I, II, and III clinical trials.
3. Studies with insufficient patient data.

### Search strategy

Two researchers searched the PubMed, Embase, and Web of Science databases up to 28 February 2023 to identify reported studies. We employed boolean logic for performing a database search, utilizing Boolean search operators such ‘AND’ and ‘OR’ to connect the search terms. The relevant retrieval strategy was as follows: (“COVID-19” OR “COVID-19”OR “SARS-CoV-2” OR “SARS CoV 2” OR “20November 19el Coronavirus* ”OR “Coronavirus Disease 2019” OR “Coronavirus Disease-2019” OR “Coronavirus Disease-19” OR “Coronavirus Disease-2019”OR “Severe Acute Respiratory Syndrome Coronavirus 2” OR “Coronavirus Disease 19” OR “Severe Acute Respiratory Syndrome Coronavirus 2” OR “SARS Coronavirus 2” OR “2019-nCoV” OR “2019 nCoV” OR “COVID-19-associated” OR “long COVID” OR “pediatric multisystem inflammatory Syndrome” OR “pulmonary intravascular coagulopathy”) AND (“vaccin*”) AND (“meta analysis” OR “meta analyses” OR “meta-analysis” OR “meta-analyses” OR “meta analysis” OR “meta analyses”). To improve the search approach, we employed the Medical Subject Headings (MeSH) database to conduct an advanced PubMed search and identify suitable MeSH terms for the mentioned search terms. Likewise, for an advanced Embase search, we utilized Emtree terms that corresponded to the specified search terms.

### Study selection and data extraction

The data extraction process followed the PRISMA 2020 guidelines, which actively guided each step of the process from the original source. Two independent authors performed a thorough screening of both abstracts and full-text articles, using predefined inclusion and exclusion criteria. For each eligible study, we documented various key details, including the first author, country of origin, journal name, publication year, study type, total number of patients, incidence rate of patients, COVID-19 vaccine types, dosage, patient demographics, laboratory results, and mortality outcomes.

Our preliminary search yielded 2,591 articles. After removing duplicates, 1,196 articles remain. Following a thorough examination of abstracts and full texts, we recognized a total of 42 systematic review studies that fulfilled our inclusion criteria (Supplementary 1).

### Evaluation of the methodological quality

A Measurement Tool to Assess Systematic Reviews 2 (AMSTAR 2) was used to evaluate the methodological quality of the included SRs. The AMSTAR 2 checklist consists of 16 items, and each item can be assessed as “Yes”, “partial Yes”, or “No”. Items 2, 4, 7, 9, 11, 13, and 15 are the key items, and the rest are non-key items. Meeting none or only one non-key item is considered high quality; meeting more than one non-key item is considered moderate quality; failing to meet one key item, with or without not meeting non-key items, is considered low quality; failing to meet more than one key item, with or without not meeting non-key items, is considered very low quality. Two researchers underwent training from an experienced researcher to utilize checklists and evaluate sample articles. Subsequently, they individually appraised these criteria for each systematic review. A meeting was conducted to deliberate on their assessments and reconcile any discrepancies. In instances where a consensus could not be achieved, a third reviewer was consulted.

### Statistical analysis

To determine the proportion of patients with myocarditis or thrombosis, we conducted a single-arm meta-analysis using random-effects models to estimate summary effects and their corresponding 95% confidence intervals (CI). The random-effects model combines the effect sizes of multiple studies, assuming that each study provides data on a distinct magnitude of impact (Lau et al., 1997). To evaluate the heterogeneity between studies, we utilized the I2 measure of inconsistency and also considered the *p*-value obtained from conducting the Cochran Q test. The I2 metric quantifies the proportion of total variance across studies attributed to heterogeneity, with values ranging from 0% to 100%. Typically, I2 values exceeding 50% suggest significant heterogeneity (Higgins et al., 2003). The statistical analyses were conducted using R version 4.1.0.

## Results

### Search results

**FIGURE 1:**
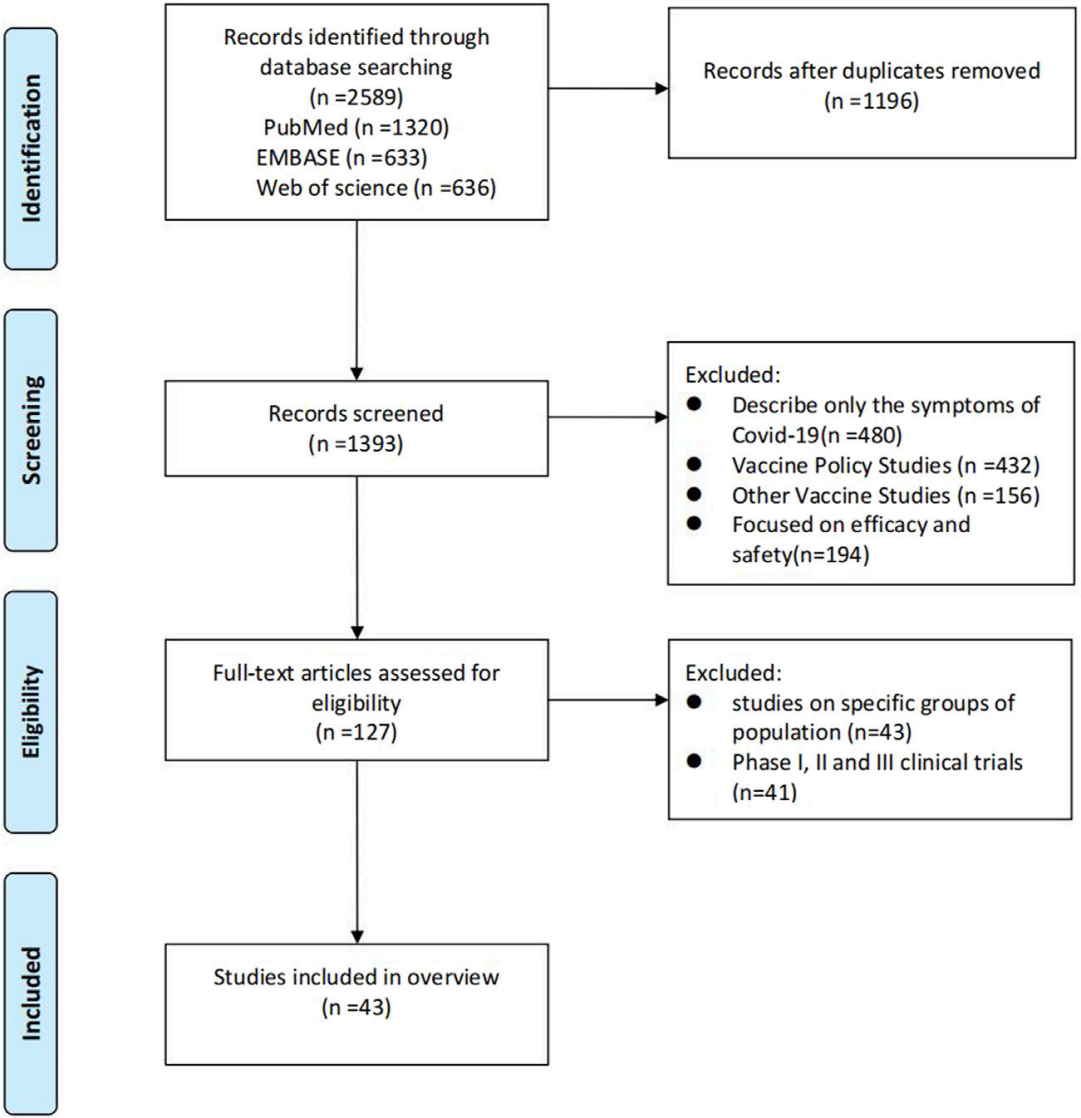
A now diagram of study screening and selection procedures is illustrated.

We conducted a comprehensive search and obtained 2,591 articles. After eliminating duplicates, we were left with 1,393 unique articles. We carefully reviewed the abstracts and full texts of each article, and excluding studies describing only the symptoms of COVID-19 (n = 480), vaccine Policy studies (n = 432), other vaccine studies (n = 156) and focused on efficacy and safety (n = 194), we have selected 127 full-text articles assessed for eligibility. and then we identified 42 systematic review studies that met our inclusion criteria (Figure 1).

### Characteristics of the included studies

A total of 42 articles were finally included. Adverse reactions following the SARSCoV-2 vaccine include myocarditis, thrombosis, cutaneous adverse reactions, fertility impairment, Guillain-Barre syndrome, dermatologic side effects, late-onset myasthenia gravis, hypermetabolic lymphadenopathy, and renal disease. Of these, thrombosis and myocarditis are the top-ranked diseases and the most dangerous (Table 1).

**TABLE 1:** Characteristics of included studies.

Author/Year of publication	Literature type	Included studies			Type of vaccine	Quality assessment	Outcome
		Study design	Number of studies	Sample size of Study (cases)			
Ahmed (2022)	SR	a	62	218	④	AMSTAR-2	Myocarditis
Castaldo (2022)	SR	b	84	1570000	②③④	NA	Headache
Hafeez (2022)	SR	a	25	69	③④	NA	TTS
Jaiswal (2022)	SR	a	25	80	③④	NA	CVST
Chou (2022)	SR meta	a,c,d	39	301	③④	NOS	Myocarditis
ElSawi (2022)	SR	a,c,d	34	460	③	NA	Thrombotic, neurological, myocarditis, etc
Franchini (2021)	SR	a	4	40	③	NA	VITT
Gao (2022)	SR meta	a,d,e	11	58620611	③④	NOS	Myocarditis, Pericarditis
Ahmed (2022)	SR	a	10	NA	③④	JBI	Takotsubo cardiomyopathy (TCM)
Kim (2022)	SR meta	a,d,e	18	664	③	NA	VITT
Kolahchi (2022)	SR	a	29	43	1 ③④	NA	VITT, AIS
Cordero (2022)	meta	g	7	17704413	④	NA	Myocarditis
Palaiodimou (2022)	SR meta	c	2	211	③	NOS	TTS–CVST
Uaprasert (2021)	SR meta	b	8	195196	①②③④	Cochrane risk-of-bias tool	Thromboembolism
Matar (2022)	SR	a	45	144	③	NA	CVST/deep vein thrombosis/pulmonary embolism
Matta (2021)	SR	a	25	16	④	NA	Myocarditis
Qaderi (2022)	SR	a,e	17	NA	②③④	NA	Cutaneous adverse reactions
Voleti (2022)	SR meta	b,e	22	58000000	②③④	NOS	Myocarditis
Pipitone (2022)	SR	b,e	7	10	③④	NA	Myocarditis
Matar (2022)	SR meta	a	75	188	④	NA	Myocarditis
Washrawirul (2022)	SR meta	a,b,e	32	946366	③④	Jadad scale/NOS	cutaneous adverse reactions
Samimisedeh (2022)	SR MA	a	102	468	①③④	JBI	Myocarditis
Shafie’ei (2022)	SR MA	e	36	680 566	②③④	JBI	Cutaneous adverse reactions
Wang (2021)	MA	e	5	217	④	NA	Myocarditis
Dorche (2021)	SR	a,c	12	54	2	NA	VITT, CVST
Salah (2021)	SR	a	8	15	③④	NA	Myocarditis
Ling (2022)	SR MA	e	22	5,792	④	JBI	Myocarditis
Zaçe (2022)	SR MA	c,d	29	NA	④	NOS	Fertility impairment
Zheng (2022)	SR MA	a,d	135	138	3 ④	NOS, JBI	Thrombotic
Kim (2022)	SR	a	7	18	③	NA	GBS
Chang (2023)	SR MA	f	42	141698	1 ③④	NOS, JBI	Myocarditis
Martora (2022)	SR	NA	183	456	③④	NA	Cutaneous Reactions
Seirafianpour (2022)	SR	a,c	180	46757	①②③④	NA	Dermatologic side effects
Triantafyllidis (2022)	SR	NA	21	57	1 ③④	ISPE, ISoP	Cutaneous Reactions
Aye (2021)	SR	a	16	36	③④	NA	Myocardial infarction and myocarditis
Avallone (2022)	SR	a	229	4,649	①③④	NA	Cutaneous manifestations
Virgilio (2023)	SR	a	18	28	③④	NA	Late-Onset Myasthenia Gravis
Dotan (2021)	SR	NA	4	42	③	NA	Thrombotic thrombocytopenia
Ling (2022)	SR MA	e	22	405272721	③	(JBI) GRADE	Myopericarditis
Treglia (2021)	SR MA	NA	9	2,354	③④	NIH	Hypermetabolic Lymph Nodes
Zhang (2022)	SR	a	90	130	①③④	NA	Renal
Lai (2022)	SR MA	b,d	17	33000000	④	NOS	Peripheral Nervous System Adverse Events
Abutaleb (2023)	SR MA	a,c,d	17	567,033,087	①③④	NIH	Cardiac arrhythmia

• Note: NOS, Newcastle–Ottawa Quality Assessment Scale; JBI, Joanna Briggs Institute’s critical appraisal tool; ISPE, the International Society for Pharmacoepidemiology; ISoP, the International Society of Pharmacovigilance; NIH, National Institutes of Health quality assessment tool; ①, inactivated vaccine; ②Recombinant protein vaccine; ③, vectors vaccine; ④, mRNA, vaccine; TTS, thrombosis with thrombocytopenia syndrome; CVST, cerebral venous sinus thrombosis; VITT, immune thrombotic thrombocytopenia; AIS, acute ischemic stroke; a, Case series/Series studies; b, RCT; c, Case–control studies/Retrospective studies; d, Cohort study; e, Observational studies/Cross-sectional study; f, Real-world studies; g, Healthcare-related databases.

### Methodological quality evaluation results

The methodological quality assessment results, conducted using the AMSTAR 2 scale for the systematic reviews included in the evaluation, are as follows: 2 systematic reviews (4.7%) were of moderate quality, 6 reviews (14.29%) were of low quality, and 34 reviews (80.95%) were of critically low quality (Table 2).

**TABLE 2:** Evaluation results of AMSTAR 2 scale.

Author (Year of publication)	Rating item																Overall quality
	1	2	3	4	5	6	7	8	9	10	11	12	13	14	15	16	
Ahmed (2022)	N	Y	Y	Y	Y	Y	Y	PY	N	N	Y	Y	N	Y	Y	Y	low
Castaldo (2022)	N	N	N	PY	Y	Y	N	Y	N	Y	Y	Y	N	N	N	Y	very low
Hafeez (2022)	Y	N	Y	Y	Y	Y	N	Y	N	N	Y	Y	N	N	Y	Y	very low
Jaiswal (2022)	Y	N	Y	Y	Y	Y	Y	Y	N	N	Y	Y	N	N	Y	Y	very low
Chou (2022)	Y	N	Y	Y	Y	Y	Y	Y	Y	N	Y	Y	Y	Y	N	Y	very low
Elsawi (2022)	Y	N	Y	Y	Y	Y	Y	Y	N	N	Y	Y	Y	Y	Y	Y	very low
Franchini (2021)	Y	N	Y	Y	N	N	N	Y	N	N	Y	Y	N	Y	Y	N	very low
Gao (2022)	Y	Y	Y	Y	Y	Y	Y	Y	Y	N	Y	Y	Y	Y	Y	Y	moderate
Ahmed (2022)	Y	Y	Y	Y	Y	Y	Y	Y	Y	N	Y	N	N	Y	Y	Y	low
Kim (2022)	Y	N	Y	N	N	N	N	Y	N	N	Y	Y	Y	Y	N	Y	very low
Kolahchi (2022)	Y	N	N	PY	Y	N	Y	Y	N	N	Y	Y	N	N	Y	Y	very low
Cordero (2022)	Y	N	Y	Y	N	N	N	Y	N	N	Y	Y	Y	Y	Y	Y	very low
Palaiodimou (2022)	Y	Y	N	Y	Y	Y	Y	Y	Y	N	Y	Y	Y	Y	Y	Y	moderate
Uaprasert (2021)	Y	Y	Y	Y	Y	Y	Y	Y	Y	N	Y	Y	Y	Y	N	Y	low
Matar (2022)	Y	N	Y	Y	Y	Y	Y	Y	Y	N	Y	Y	Y	Y	N	Y	low
Matta (2021)	Y	PY	Y	Y	Y	Y	N	Y	N	Y	Y	Y	N	N	N	Y	very low
Qaderi (2022)	N	N	Y	Y	Y	Y	N	Y	N	N	Y	Y	N	N	N	Y	very low
Voleti (2022)	Y	N	Y	Y	Y	Y	N	Y	Y	Y	Y	Y	Y	Y	Y	Y	very low
Pipitone (2022)	N	N	Y	PY	N	N	N	Y	N	Y	Y	Y	N	N	N	Y	very low
Matar (2022)	Y	N	Y	Y	Y	Y	N	Y	Y	Y	Y	N	N	N	N	Y	very low
Washrawirul (2022)	Y	N	Y	PY	Y	Y	N	Y	Y	Y	Y	N	N	N	N	Y	very low
Samimisedeh (2022)	Y	N	Y	Y	Y	Y	N	Y	Y	N	Y	N	N	N	N	N	very low
Shafie’ei (2022)	Y	N	Y	PY	Y	Y	N	Y	Y	N	Y	N	N	Y	Y	N	very low
Wang (2021)	N	N	Y	PY	N	N	N	N	N	N	Y	Y	N	N	Y	N	very low
Dorche (2021)	N	N	N	PY	N	N	N	Y	N	N	Y	Y	N	N	Y	Y	very low
Salah (2021)	Y	N	Y	PY	N	N	N	Y	N	N	Y	Y	N	N	Y	Y	very low
Ling (2022)	Y	Y	Y	Y	N	N	N	Y	Y	Y	Y	Y	Y	Y	Y	Y	low
Zaçe (2022)	Y	N	Y	PY	Y	Y	N	Y	Y	N	Y	Y	Y	Y	Y	Y	very low
Zheng (2022)	N	N	Y	Y	Y	Y	N	Y	Y	Y	Y	N	N	N	N	Y	very low
Kim (2022)	Y	N	Y	PY	Y	Y	N	Y	Y	Y	Y	Y	N	N	Y	Y	very low
Chang (2023)	N	Y	Y	Y	Y	Y	N	Y	Y	N	Y	Y	Y	Y	Y	Y	low
Martora (2022)	N	N	Y	Y	N	N	N	Y	N	N	Y	Y	N	N	N	Y	very low
Seirafianpour (2022)	Y	N	Y	Y	Y	Y	N	Y	N	N	Y	Y	N	N	Y	Y	very low
Triantafyllidis (2022)	N	N	Y	PY	Y	Y	N	Y	Y	N	Y	Y	N	N	Y	Y	very low
Aye (2021)	Y	Y	Y	Y	N	N	N	Y	N	Y	Y	Y	N	N	Y	Y	very low
Avallone (2022)	N	N	Y	Y	N	N	N	Y	N	N	Y	Y	N	N	Y	Y	very low
Virgilio (2023)	N	N	Y	PY	Y	N	N	Y	N	Y	Y	Y	N	N	Y	Y	very low
Dotan (2021)	N	N	N	PY	N	N	N	Y	N	Y	Y	Y	N	N	Y	N	very low
Treglia (2021)	N	N	N	PY	Y	Y	N	Y	N	Y	Y	N	N	N	Y	Y	very low
Zhang (2022)	Y	N	Y	PY	N	N	N	Y	N	N	Y	Y	N	N	Y	Y	very low
Lai (2022)	N	Y	Y	Y	Y	Y	N	Y	Y	Y	Y	Y	N	N	Y	Y	very low
Abutaleb (2023)	Y	Y	Y	Y	Y	Y	N	Y	Y	Y	Y	N	N	N	N	Y	very low

Note: Y. yes; PY., partially yes; N. no; Item 1: Are research questions and inclusion criteria based on PICO; Item 2: Is it stated that the research methods of the systematic review were determined before implementation, and are inconsistencies with the research protocol explained; Item 3: Is the type of included studies indicated when including literature; Item 4: Is a comprehensive search strategy adopted; Item 5: Is double screening used for literature selection; Item 6: Is double data extraction done by two independent reviewers; Item 7: Is an excluded studies list provided with reasons; Item 8: Are included studies described in detail; Item 9: Is an appropriate tool used to assess the risk of bias for each included study; Item 10: Are funding sources reported for each included study; Item 11: Are the statistical methods used for combining study results in meta-analysis appropriate; Item 12: Is the potential impact of bias risk of individual studies on the results of meta-analysis or other evidence synthesis assessed during meta-analysis; Item 13: When interpreting or discussing results, is the bias risk of included studies considered; Item 14: Is reasonable explanation and discussion provided for the heterogeneity in the studies; Item 15: Is publication bias thoroughly investigated and its potential impact on results discussed; Item 16: Are any potential sources of conflicts of interest reported. Items 2, 4, 7, 9, 11, 13, and 15 are the key items, and the rest are non-key items. Meeting none or only one non-key item is considered high quality; meeting more than one non-key item is considered moderate quality; failing to meet one key item, with or without not meeting non-key items, is considered low quality; failing to meet more than one key item, with or without not meeting non-key items, is considered very low quality.

### The risk of myocarditis and thromboembolism after vaccination

Twenty-five studies reported on the eventuality of unfavorable responses following different types of vaccinations, among which the SARSCoV-2 vaccine was mainly included inactivated, recombinant protein, vectors vaccine and mRNA vaccines. Six articles analyzed vaccine-induced myocarditis while ten articles examined thrombosis caused by different types of vaccines.

**FIGURE 2:**
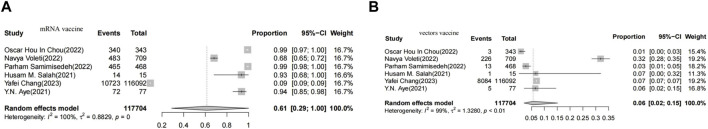
Forest plot presenting the association of vaccine-associated myocarditis with vector-based vaccines **(A)**, Forest plot presenting the association of vaccine-associated myocarditis with non-vector-ba sed vaccines **(B)**.

In cases of myocarditis, the pooled proportion of myocarditis after Vector vaccination was 6% (CI 2-15), while the pooled proportion after mRNA vaccination was 61% (CI 29-100) (Figure 2).

**FIGURE 3:**
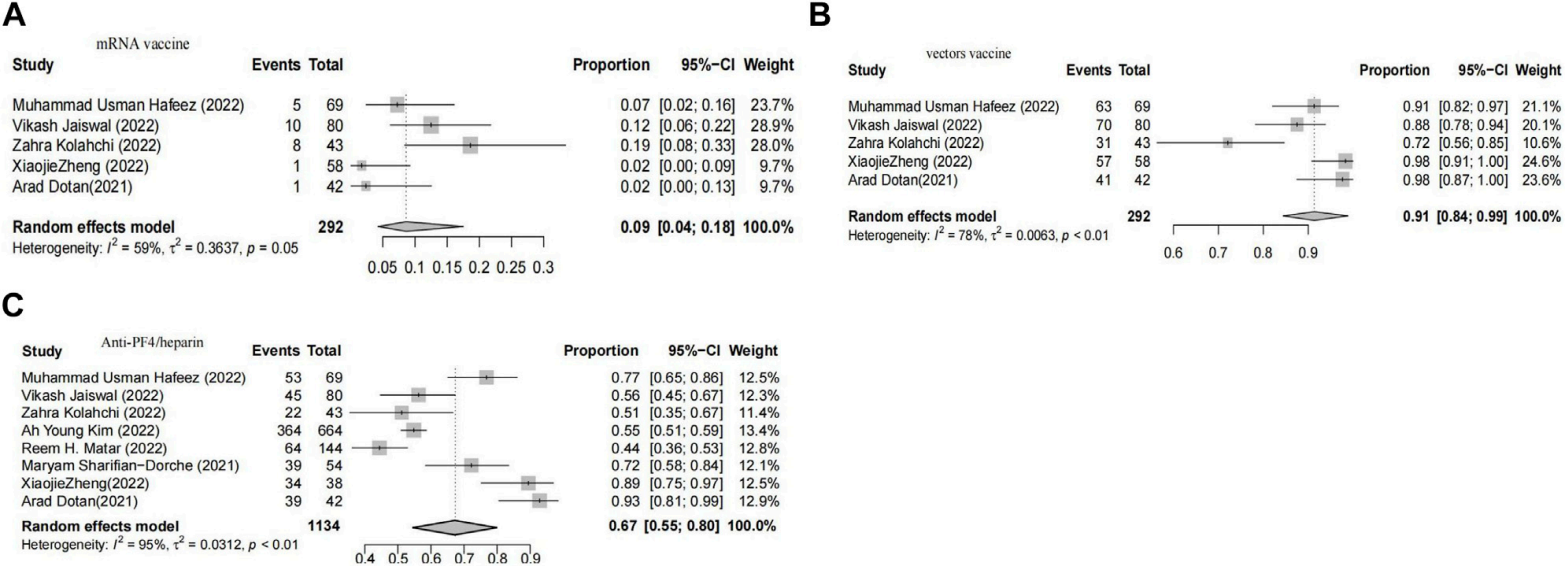
Forest plot presenting the association of vaccine-associated thrombosis with vector-based vaccines **(A)**, Forest plot presenting the association of vaccine-associated thrombosis with non -vector-based vaccines **(B)**, Forest plot of meta-analysis to estimate the proportion of patients with positive antiplatelet factor 4 antibody test **(C)**.

In cases of thrombosis, the pooled proportion of myocarditis after Vector vaccination was 91% (95% CI 84-99), while the pooled proportion of myocarditis after mRNA vaccination was 9% (95% CI 4-18). The meta-analysis included eight studies that assessed the anti-PF4 antibody test, revealing a positivity rate of 67% (95% CI 55–80) (Figure 3).

**FIGURE 4:**
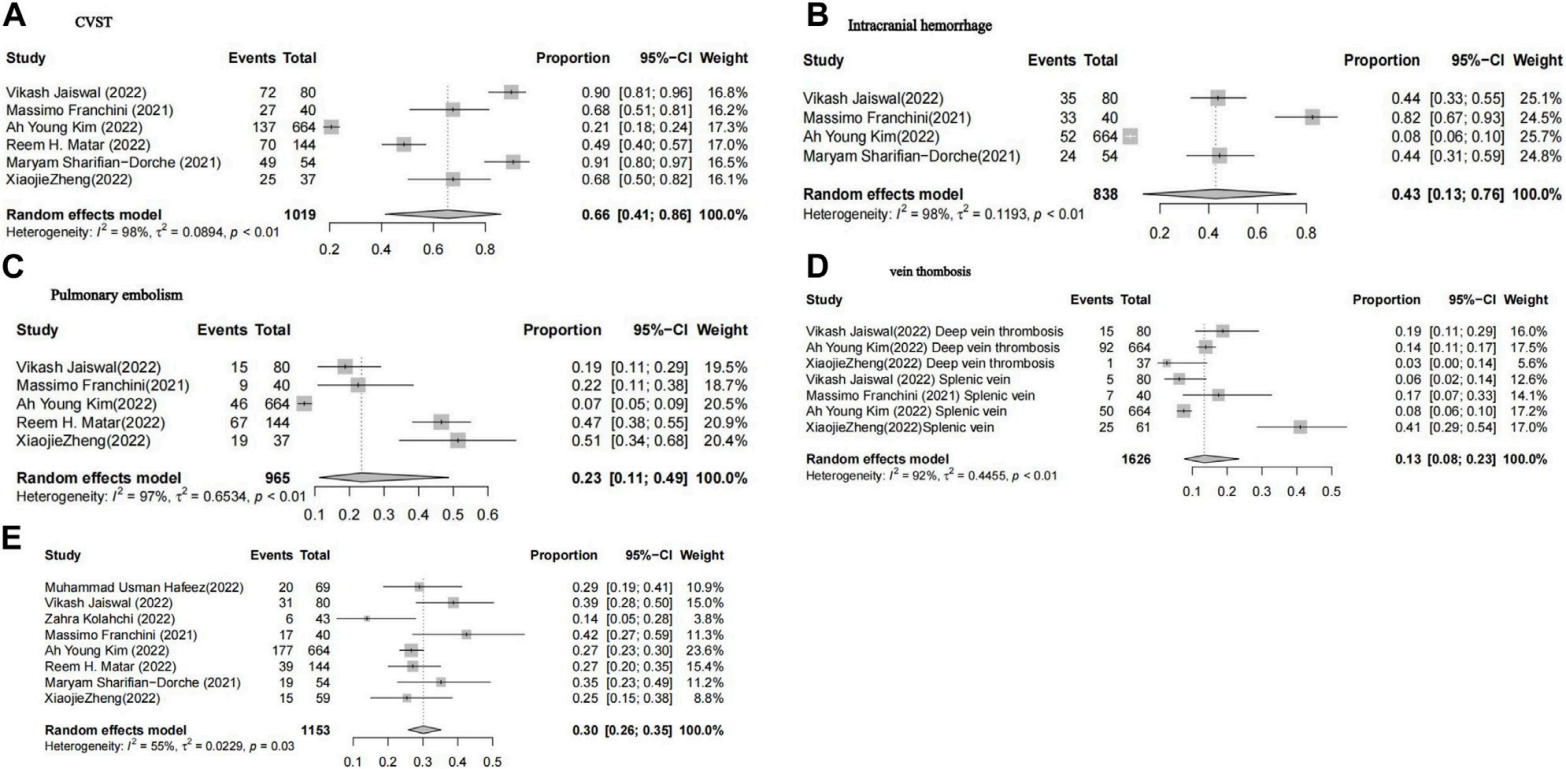
Forest plot of meta-ana lysis lo estimate the proportion of cvst, Intracranial hemorrhage, vein thombosis and Pulmonary embolism in a ll pa tients with vaccine-induced im mune thrombotic tbrombocytopenia **(A–D)**, Forest plot of meta-analysis to estimate the overall, mortality rate of patients with vaccine-induced immune thrombotic **(E)**.

Among patients diagnosed with vaccine-induced immune thrombotic thrombocytopenia (VITT), a meta-analysis found that 66% experienced cerebral venous sinus thrombosis (CVST) (Figure 4A), while 43% experienced intracranial hemorrhage (ICH) (Figure 4B). The analysis revealed that the combined incidence rates of deep vein thrombosis and pulmonary embolism among VITT patients were 13% and 23%, respectively (Figure 4C,D).

The overall case fatality rate following vaccination was estimated to be at least 30% [95% CI:26-35; I2 = 55%] (Figure 4E).

## Discussion

This study offers a comprehensive analysis of adverse outcomes following SARS-CoV-2 vaccination, based on a systematic review of studies published up to February 2023. We found that the most prevalent adverse effects include myocarditis, thrombotic events, skin reactions, potential fertility issues, Guillain-Barre Syndrome, dermatologic side effects, late-onset myasthenia gravis, hypermetabolic lymph nodes, and renal complications.

Our analysis highlights that reactions at the injection site, particularly cutaneous effects, are common with COVID-19 vaccines. These reactions are generally self-limiting and rarely require extensive treatment (Qaderi et al., 2022). Interestingly, outcomes like fertility impairment, late-onset myasthenia gravis, hypermetabolic lymph nodes, and renal issues are infrequent and not well-documented in the vaccinated population. The studies we reviewed did not provide substantial evidence of causality, underscoring the need for larger, more comprehensive studies (Virgilio et al., 2022).

We observed that thrombosis and myocarditis were among the most severe adverse outcomes. The incidence of myocarditis was approximately 3.7 per 100,000 vaccine doses, a rarity compared to historical data from the Vaccine Adverse Event Reporting System (VAERS), which reported a myocarditis incidence of 0.1% between 1990 and 2018 (Kim et al., 2022a; Cordero et al., 2022). In our overview, vaccine-associated thrombosis data were limited, with only one study reporting incidence rates post-vaccination with ChAdOx1 nCoV-19 and Ad26.COV2. S (Kim et al., 2022a). Notably, thrombotic thrombocytopenia has been documented in the history of various vaccinations, such as those for H1N1, rabies, and pneumococcal disease (Bansal et al., 2021; Kim et al., 2022b; Kow et al., 2022). The emergence of vaccine-induced immune thrombotic thrombocytopenia (VITT), particularly associated with viral vector COVID-19 vaccines, is a significant development.

A further comparative analysis on myocarditis or thrombotic events in mRNA vs vector-based vaccines revealed notable differences. The myocarditis rate post-vector vaccines was 6%, in contrast to 61% post-mRNA vaccines. Safety concerns regarding mRNA and vector vaccines have been the subject of debate, with reports of myocarditis following mRNA COVID-19 vaccinations by the CDC and VAERS [(Oster et al., 2022), (Patone et al., 2022), (Nygaard et al., 2022)]. The autoimmune response hypothesis is one potential explanation for vaccine-induced myocarditis, with ongoing research into the role of immune reactions, antibody cross-reactivity, and hormonal fluctuations (Kouhpayeh and Ansari, 2022; Chang et al., 2023).

Conversely, our data showed that 91% of thrombosis cases were associated with vector vaccines, aligning with CDC reports on the rarity of adverse reactions in these vaccines. The WHO’s diagnostic algorithm helped establish a causal link in rare cases of immune thrombocytopenia mediated by platelet factor 4 (PF4) antibodies post-vaccination with ChAdOx1 nCov-19 (Pomara et al., 2021). VITT pathophysiology seems connected to interactions between free DNA and PF4 in adenovirus-based vaccines, leading to PF4-reactive antibodies (Greinacher et al., 2021; Kennedy et al., 2021). This study found a 67% positivity rate for anti-PF4 antibodies in a meta-analysis of eight studies, suggesting an autoimmune mechanism similar to heparin-induced thrombocytopenia.

In thrombosis cases, cerebral venous sinus thrombosis (CVST) was most common, occurring in 66% of cases, followed by intracranial hemorrhage, pulmonary embolism, and vein thrombosis. The symptoms of CVST, depending on the affected vein or sinus, can mimic various neurological conditions (Sharifian-Dorche et al., 2021). The fatality rate among VITT patients is alarmingly high at 30%, highlighting the need for prompt recognition and management of CVST and VITT post-vaccination (Shafie et al., 2022; Fengwen et al., 2022; Pothiawala et al., 2022; Shuangming, 2023).

### Limitations

This overview has certain limitations, including incomplete case descriptions in some of the reviewed case reports and series. Additionally, the analysis primarily relies on case reports and series, which inherently provide a smaller scale of evidence in comparison to more extensive clinical studies. As a result, the conclusions that can be drawn from this research may be somewhat restricted. Publication bias was not evaluated in this study, as the included studies in the proportionality meta-analysis were not considered comparable.

## Conclusion

The findings of this overview suggest that while most adverse reactions to vaccination are self-limiting and require minimal or no treatment, rare outcomes like myocarditis and thrombosis can be severe and potentially life-threatening. Thus, prompt identification and appropriate therapy are crucial for improving outcomes in affected individuals. Comparative analysis reveals differing patterns of adverse events between mRNA and vector-based vaccines. Further research into the pathophysiological mechanisms of PF4-reactive antibodies in VITT cases is warranted. Timely recognition and management of VITT-related complications, particularly cerebral venous sinus thrombosis, are paramount to reduce morbidity and mortality. In conclusion, COVID-19 vaccination is vital for pandemic control, but continuous surveillance, research, and proactive management of adverse events are imperative for ensuring the safety and efficacy of global vaccination efforts.

## Data Availability

The original contributions presented in the study are included in the article/Supplementary material, further inquiries can be directed to the corresponding author.
