# Supplementary material for: An overview and single-arm meta-analysis of immune-mediated adverse events following COVID-19 vaccination
Source: Front Pharmacol. 2024 Jun 12;15:1308768. doi: 10.3389/fphar.2024.1308768 (PMC11200080; doi:10.3389/fphar.2024.1308768)
Supplement: Supplementary file 1 [file DataSheet1.docx]

Supplementary1

[1]Kim AY, Woo W, Yon DK, Lee SW, Yang JW, Kim JH, etc. Thrombosis patterns and clinical outcome of COVID-19 vaccine-induced immune thrombotic thrombocytopenia: A Systematic Review and Meta-Analysis. Int J Infect Dis 2022 ;119:130-139. doi: 10.1016/j.ijid.2022.03.034.

[2]Kolahchi Z, Khanmirzaei M, Mowla A. Acute ischemic stroke and vaccine-induced immune thrombotic thrombocytopenia post COVID-19 vaccination; a systematic review. J Neurol Sci 2022;439:120327. doi: 10.1016/j.jns.2022.120327.

[3]ElSawi HA, Elborollosy A. Immune-mediated adverse events post-COVID vaccination and types of vaccines: a systematic review and meta-analysis. Egypt J Intern Med2022;34:44. doi: 10.1186/s43162-022-00129-5.

[4]Zaçe D, La Gatta E, Petrella L, Di Pietro ML. The impact of COVID-19 vaccines on fertility-A systematic review and meta-analysis. Vaccine 2022;40:6023-6034. doi: 10.1016/j.vaccine.2022.09.019.

[5]Castaldo M, Waliszewska-Prosół M, Koutsokera M, Robotti M, Straburzyński M, Apostolakopoulou L, et al. Headache onset after vaccination against SARS-CoV-2: a systematic literature review and meta-analysis. J Headache Pain 2022 ;23:41. doi: 10.1186/s10194-022-01400-4.

[6]Chen J, Cai Y, Chen Y, Williams AP, Gao Y, Zeng J. Nervous and Muscular Adverse Events after COVID-19 Vaccination: A Systematic Review and Meta-Analysis of Clinical Trials. Vaccines (Basel) 2021 ;9:939. doi: 10.3390/vaccines9080939.

[7]Hafeez MU, Ikram M, Shafiq Z, Sarfraz A, Sarfraz Z, Jaiswal V, et al. COVID-19 Vaccine-Associated Thrombosis With Thrombocytopenia Syndrome (TTS): A Systematic Review and Post Hoc Analysis. Clin Appl Thromb Hemost. 2021 ;27. doi: 10.1177/10760296211048815.

[8]Jaiswal V, Nepal G, Dijamco P, Ishak A, Dagar M, Sarfraz Z, etc. Cerebral Venous Sinus Thrombosis Following COVID-19 Vaccination: A Systematic Review. J Prim Care Community Health 2022 ;13. doi: 10.1177/21501319221074450.

[9]Chou OHI, Mui J, Chung CT, Radford D, Ranjithkumar S, Evbayekha E, etc. COVID-19 vaccination and carditis in children and adolescents: a systematic review and meta-analysis. Clin Res Cardiol2022;111:1161-1173. doi: 10.1007/s00392-022-02070-7.

[10]Franchini M, Liumbruno GM, Pezzo M. COVID-19 vaccine-associated immune thrombosis and thrombocytopenia (VITT): Diagnostic and therapeutic recommendations for a new syndrome. Eur J Haematol 2021 ;107:173-180. doi: 10.1111/ejh.13665.

[11]Gao J, Feng L, Li Y, Lowe S, Guo Z, Bentley R, etc. A Systematic Review and Meta-analysis of the Association Between SARS-CoV-2 Vaccination and Myocarditis or Pericarditis. Am J Prev Med 2023;64(2):275-284. doi: 10.1016/j.amepre.2022.09.002.

[12]Khalid Ahmed S, Gamal Mohamed M, Abdulrahman Essa R, Abdelaziz Ahmed Rashad Dabou E, Omar Abdulqadir S, Muhammad Omar R. Global reports of takotsubo (stress) cardiomyopathy following COVID-19 vaccination: A systematic review and meta-analysis. Int J Cardiol Heart Vasc. 2022;43:101108. doi: 10.1016/j.ijcha.2022.101108.

[13]Treglia G, Cuzzocrea M, Giovanella L, Elzi L, Muoio B. Prevalence and Significance of Hypermetabolic Lymph Nodes Detected by 2-[18F]FDG PET/CT after COVID-19 Vaccination: A Systematic Review and a Meta-Analysis. Pharmaceuticals (Basel). 2021;14:762. doi: 10.3390/ph14080762.

[14]Cordero A, Cazorla D, Escribano D, Quintanilla MA, López-Ayala JM, Berbel PP, etc. Myocarditis after RNA-based vaccines for coronavirus. Int J Cardiol. 2022;353:131-134. doi: 10.1016/j.ijcard.2022.01.037.

[15]Palaiodimou L, Stefanou MI, de Sousa DA, Coutinho JM, Papadopoulou M, Papaevangelou V, et al. Cerebral venous sinus thrombosis in the setting of COVID-19 vaccination: a systematic review and meta-analysis. J Neurol. 2022 ;269:3413-3419. doi: 10.1007/s00415-022-11101-2.

[16]Uaprasert N, Panrong K, Rojnuckarin P, Chiasakul T. Thromboembolic and hemorrhagic risks after vaccination against SARS-CoV-2: a systematic review and meta-analysis of randomized controlled trials. Thromb J 2021;19(1):86. doi: 10.1186/s12959-021-00340-4.

[17]Matar RH, Than CA, Nakanishi H, Daniel RS, Smayra K, Sim BL, etc. Outcomes of patients with thromboembolic events following coronavirus disease 2019 AstraZeneca vaccination: a systematic review and meta-analysis. Blood Coagul Fibrinolysis 2022 ;33:90-112. doi: 10.1097/MBC.0000000000001113.

[18]Medeiros KS, Costa APF, Sarmento ACA, Freitas CL, Gonçalves AK. Side effects of COVID-19 vaccines: a systematic review and meta-analysis protocol of randomised trials. BMJ Open 2022 Feb 24;12:e050278. doi: 10.1136/bmjopen-2021-050278.

[19]Pischel L, Patel KM, Goshua G, Omer SB. Adenovirus-Based Vaccines and Thrombosis in Pregnancy: A Systematic Review and Meta-analysis. Clin Infect Dis 2022 ;75:1179-1186. doi: 10.1093/cid/ciac080.

[20]Matta A, Kunadharaju R, Osman M, Jesme C, McMiller Z, Johnson EM, etc. Clinical Presentation and Outcomes of Myocarditis Post mRNA Vaccination: A Meta-Analysis and Systematic Review. Cureus 2021 ;13:e19240. doi: 10.7759/cureus.19240.

[21]Qaderi K, Golezar MH, Mardani A, Mallah MA, Moradi B, Kavoussi H, etc. Cutaneous adverse reactions of COVID-19 vaccines: A systematic review. Dermatol Ther 2022 ;35:e15391. doi: 10.1111/dth.15391.

[22]Ling RR, Ramanathan K, Tan FL, Tai BC, Somani J, Fisher D, etc. Myopericarditis following COVID-19 vaccination and non-COVID-19 vaccination: a systematic review and meta-analysis. Lancet Respir Med 2022 ;10:679-688. doi: 10.1016/S2213-2600(22)00059-5.

[23]Chang Y, Lv G, Liu C, Huang E, Luo B. Cardiovascular safety of COVID-19 vaccines in real-world studies: a systematic review and meta-analysis. Expert Rev Vaccines2023;22:25-34. doi: 10.1080/14760584.2023.2150169.

[24]Virgilio E, Tondo G, Montabone C, Comi C. COVID-19 Vaccination and Late-Onset Myasthenia Gravis: A New Case Report and Review of the Literature. Int J Environ Res Public Health 2022 ;20:467. doi: 10.3390/ijerph20010467.

[25]Sharifian-Dorche M, Bahmanyar M, Sharifian-Dorche A, Mohammadi P, Nomovi M, Mowla A. Vaccine-induced immune thrombotic thrombocytopenia and cerebral venous sinus thrombosis post COVID-19 vaccination; a systematic review. J Neurol Sci 2021 ;428:117607. doi: 10.1016/j.jns.2021.117607.

[26]Shafie'ei M, Jamali M, Akbari Z, Sarvipour N, Ahmadzade M, Ahramiyanpour N. Cutaneous adverse reactions following COVID-19 vaccinations: A systematic review and meta-analysis. J Cosmet Dermatol 2022;21:3636-3650. doi: 10.1111/jocd.15261.

[27]Voleti N, Reddy SP, Ssentongo P. Myocarditis in SARS-CoV-2 infection vs. COVID-19 vaccination: A systematic review and meta-analysis. Front Cardiovasc Med2022;9:1314. doi: 10.3389/fcvm.2022.951314.

[28]Matar RH, Mansour R, Nakanishi H, Smayra K, El Haddad J, Vankayalapati DK, etc. Clinical Characteristics of Patients with Myocarditis following COVID-19 mRNA Vaccination: A Systematic Review and Meta-Analysis. J Clin Med2022;11:4521. doi: 10.3390/jcm11154521.

[29]Washrawirul C, Triwatcharikorn J, Phannajit J, Ullman M, Susantitaphong P, Rerknimitr P. Global prevalence and clinical manifestations of cutaneous adverse reactions following COVID-19 vaccination: A systematic review and meta-analysis. J Eur Acad Dermatol Venereol2022;36:1947-1968. doi: 10.1111/jdv.18294. [30]Samimisedeh P, Jafari Afshar E, Shafiabadi Hassani N, Rastad H. Cardiac MRI Findings in COVID-19 Vaccine-Related Myocarditis: A Pooled Analysis of 468 Patients. J Magn Reson Imaging2022;56:971-982. doi: 10.1002/jmri.28268.

[31]Wang M, Wen W, Zhou M, Wang C, Feng ZH. Meta-Analysis of Risk of Myocarditis After Messenger RNA COVID-19 Vaccine. Am J Cardiol2022;167:155-157. doi: 10.1016/j.amjcard.2021.12.007.

[32]Salah HM, Mehta JL. COVID-19 Vaccine and Myocarditis. Am J Cardiol 2021;157:146-148. doi: 10.1016/j.amjcard.2021.07.009.

[33]Zheng X, Gao F, Wang L, Meng Y, Ageno W, Qi X. Incidence and outcomes of splanchnic vein thrombosis after diagnosis of COVID-19 or COVID-19 vaccination: a systematic review and meta-analysis. J Thromb Thrombolysis2023;55:18-31. doi: 10.1007/s11239-022-02732-3.

[34]Martora F, Battista T, Marasca C, Genco L, Fabbrocini G, Potestio L. Cutaneous Reactions Following COVID-19 Vaccination: A Review of the Current Literature. Clin Cosmet Investig Dermatol2022;15:2369-2382. doi: 10.2147/CCID.S388245.

[35]Seirafianpour F, Pourriyahi H, Gholizadeh Mesgarha M, Pour Mohammad A, Shaka Z, Goodarzi A. A systematic review on mucocutaneous presentations after COVID-19 vaccination and expert recommendations about vaccination of important immune-mediated dermatologic disorders. Dermatol Ther2022;35:15461.doi: 10.1111/dth.15461.

[36]Triantafyllidis KK, Giannos P, Stathi D, Kechagias KS. Graves' disease following vaccination against SARS-CoV-2: A systematic review of the reported cases. Front Endocrinol (Lausanne)2022;13:938001. doi: 10.3389/fendo.2022.938001.

[37]Aye YN, Mai AS, Zhang A, Lim OZH, Lin N, Ng CH, etc. Acute myocardial infarction and myocarditis following COVID-19 vaccination. QJM2023 ;116:279-283. doi: 10.1093/qjmed/hcab252.

[38]Avallone G, Quaglino P, Cavallo F, Roccuzzo G, Ribero S, Zalaudek I, etc. SARS-CoV-2 vaccine-related cutaneous manifestations: a systematic review. Int J Dermatol2022;61:1187-1204. doi: 10.1111/ijd.16063.

[39]Dotan A, Shoenfeld Y. Perspectives on vaccine induced thrombotic thrombocytopenia. J Autoimmun2021;121:102663. doi: 10.1016/j.jaut.2021.102663.

[40]Zhang J, Cao J, Ye Q. Renal Side Effects of COVID-19 Vaccination. Vaccines (Basel) 2022;10:1783. doi: 10.3390/vaccines10111783.

[41]Lai YH, Chen HY, Chiu HH, Kang YN, Wong SB. Peripheral Nervous System Adverse Events after the Administration of mRNA Vaccines: A Systematic Review and Meta-Analysis of Large-Scale Studies. Vaccines (Basel)2022 ;10:2174. doi: 10.3390/vaccines10122174.

[42]Abutaleb MH, Makeen HA, Meraya AM, Alqahtani SS, Al-Mass BI, Aljazaeri RO, etc. Risks of Cardiac Arrhythmia Associated with COVID-19 Vaccination: A Systematic Review and Meta-Analysis. Vaccines (Basel)2023;11:112. doi: 10.3390/vaccines11010112.

[43]Pipitone G, Rindi LV, Petrosillo N, Foti NAM, Caci G, Iaria C, etc. Vaccine-Induced Subacute Thyroiditis (De Quervain's) after mRNA Vaccine against SARS-CoV-2: A Case Report and Systematic Review. Infect Dis Rep2022;14:142-154. doi: 10.3390/idr14010018.
